# Challenges in the diagnosis of systemic lupus erythematosus^✰^

**DOI:** 10.1016/j.clinme.2025.100509

**Published:** 2025-09-04

**Authors:** Jean Scopes, Md Yuzaiful Md Yusof

**Affiliations:** a Rheumatology Department, Chapel Allerton Hospital, Leeds Teaching Hospitals NHS Trust, Leeds, UK; b Leeds Institute of Rheumatic and Musculoskeletal Medicine, University of Leeds, Chapel Allerton Hospital, Chapeltown Road, Leeds LS7 4SA, UK; c NIHR Leeds Biomedical Research Centre, Leeds Teaching Hospitals NHS Trust, Leeds, UK

**Keywords:** Classification, Diagnosis, Systemic lupus erythematosus

## Abstract

Systemic lupus erythematosus (SLE) is a lifelong, complex, multi-system, autoimmune condition which can occur at any age, most commonly in female adults in their reproductive years. Diagnosis is often delayed, with reported time from symptom onset to diagnosis as long as 6 years. Delayed diagnosis can result in irreversible organ damage, acute hospital admission, poor health-related outcomes and increased risk of mortality. SLE is a clinical diagnosis that requires a combination of clinical and immunological features attributable to lupus. Classification criteria have undergone several revisions and can be used to guide diagnosis. This article will review challenges in making a timely diagnosis of SLE, including heterogeneity of clinical and immunological features, disease mimickers, problems with diagnostic tests, and difficulties faced by general physicians and rheumatologists, with the aim to update knowledge among general physicians on clinical presentations and when to refer suspected patients to specialists.

## Highlights

• A diagnosis of systemic lupus erythematosus (SLE) requires a combination of clinical and immunological features attributed to lupus.
• Many early symptoms may be non-specific, thus contributing to a delay in diagnosis.
• Antinuclear antibody (ANA) is a key diagnostic test, but it has very low specificity.
• When evaluating for SLE, to consider mimickers including viral infections, malignancy, side effects from drugs and comorbidities.
• Improved awareness and better diagnostics in the future may facilitate timely diagnosis and improve long-term outcomes.

## Introduction

Systemic lupus erythematosus (SLE) is a chronic autoimmune disease that causes inflammation and subsequent damage to a range of organs including skin, joints, and other major organs such as the kidneys, brain, lungs and heart. The exact aetiology of SLE remains unknown, although combinations of genetic, environmental, immunological and hormonal factors appear to play a key role. The course of SLE is typically characterised by periods of relapse and remission following treatment with immunosuppressive therapies.

SLE can present at any age, with 15–20% of patients developing it during childhood. Adult women are nine times more likely to be affected than men, with a peak incidence in the reproductive years.^1^ This sex difference is less marked in childhood, especially pre-pubertal. Incidence rates of SLE in Europe, North and South America are estimated between 1 and 23 per 100,000 annually.^2^ In the UK, SLE affects approximately 1 in 1,000 adults.^3^ It is most frequently observed in people of African-Caribbean and South Asian ancestry.^3^ In these ancestral groups, SLE appears more severe compared to patients of European ancestry, with a higher incidence of renal involvement in particular.^4^ Juvenile-onset SLE (JSLE) has a more aggressive presentation compared to adult-onset SLE, including renal and neurological complications.^5^ Although SLE is uncommon, it has substantial unmet needs since mortality is increased by 18-fold in JSLE and threefold in adult-onset SLE compared to the general population.^5^^,^^6^ Standardised mortality ratio is highest in the first year of SLE diagnosis, at 5.4.^7^ This is attributed to the delay from symptom onset to diagnosis, which is often as long as 6.4 years.^8^ This delay may occur in both primary and secondary care, as evidenced by increased number of GP presentations prior to diagnosis^9^ and the time taken for internists to refer patients to rheumatologists.^10^ Diagnostic delay can lead to increased disease activity and organ damage, and significantly impair quality of life and long-term health outcomes.^11^

## Diagnosis and classification criteria

SLE is heterogenous in terms of clinical features. Owing to the complexity of clinical presentation, diagnosis is made clinically, by experienced clinicians. This requires a combination of clinical and immunological features attributable to SLE. A complete medical history, as well as relevant clinical and laboratory findings including urine dipstick and urinalysis, are required for assessment of SLE.

Diagnosis can be guided by classification criteria. Classification criteria are different from diagnostic criteria. The former are used primarily to group patients for research, while diagnostic criteria are used to diagnose patients in clinical practice. Thus, classification criteria are often stricter than diagnostic criteria and have high specificity (ie they have a low rate of false positive). Consequently, some common features that are less specific for SLE (Raynaud’s and anti-Ro antibody) and uncommon features (lupus hepatitis, lupus enteritis, shrinking lung syndrome, myocarditis and ophthalmic lupus) are omitted in classification criteria. Moreover, patients with SLE often present with a high burden of less specific symptoms such as fatigue and myalgia, but these symptoms do not assist or inform diagnosis.

Classification criteria may be updated over time. Current classification criteria are the 2019 European Alliance of Associations for Rheumatology (EULAR)–American College of Rheumatology (ACR) (Table 1). There are two main differences from the previous criteria: a) patients must have anti-nuclear antibody (ANA)-positive (≥1:80 titre) at least once, and b) each criterion is assigned a weight of 2–10, where only the highest item of each domain is counted. Criteria do not have to be present simultaneously and they must be attributed to SLE. Within each domain, only the highest-weighted criterion contributes towards the total score. Patients can be classified as SLE if fulfilling a score of 10 or more, and at least one clinical criterion is fulfilled.^12^

**Table 1:** The 2019 EULAR-ACR classification criteria for SLE.

Patients are eligible for these criteria only if they have a positive ANA test ≥1:80 (at least once). SLE can be classified if meeting ≥10 total points		
Domain	Manifestation	Points
Constitutional	Fever	2
Cutaneous	Non-scarring alopecia	2
	Oral ulcers	2
	Subacute cutaneous/discoid lupus	4
	Acute cutaneous lupus	6
Arthritis	Swelling or effusion or tenderness in ≥ 2 joints and ≥ 30 min morning stiffness	6
Neuropsychiatric	Delirium	2
	Psychosis	3
	Seizure (generalised or partial/focal)	5
Serositis	Pleural or pericardial effusion	5
	Acute pericarditis	6
Hematologic	Leucopenia (white cell count < 4 × 10^9^/L)	3
	Thrombocytopenia (platelet count < 100 × 10^9^/L)	4
	Autoimmune haemolysis	4
Renal	Proteinuria > 0.5 g in 24 hrs	4
	Class II or V lupus nephritis	8
	Class III or IV lupus nephritis	10
Antiphospholipid antibody	Anticardiolipin IgG > 40 iU/mL	2
	Anti-β2 glycoprotein IgG > 40 iU/mL	2
	Lupus anticoagulant	2
Complement	Low C3 or C4	3
	Low C3 and C4	4
Highly specific antibodies	Anti-dsDNA	6
	Anti-Smith (Sm)	6

### Challenges in SLE diagnosis

**Fig. 1:**
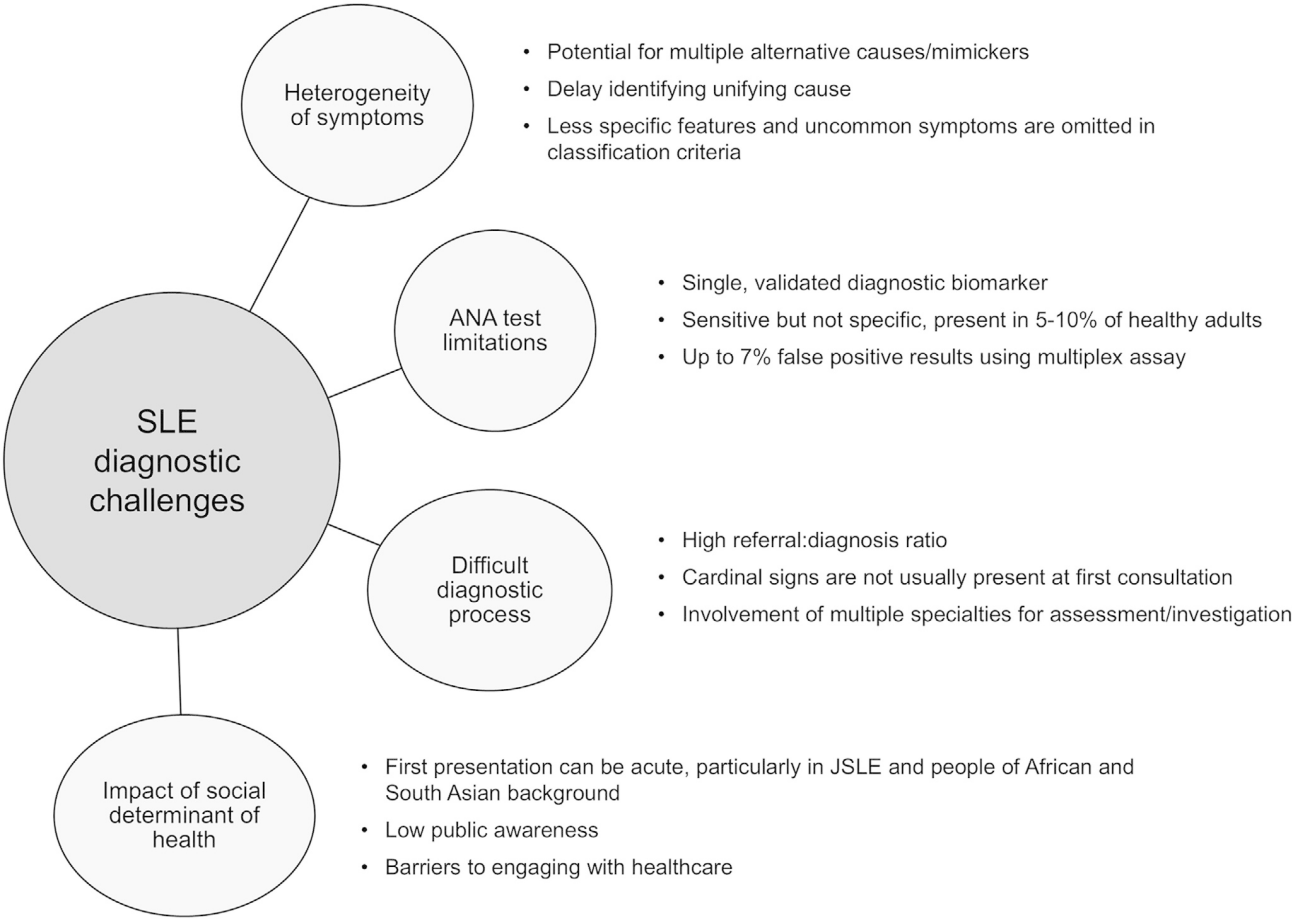
Diagnostic challenges in systemic lupus erythematosus.

There are various challenges in SLE diagnosis. These are summarised in Fig. 1.

### Problems with ANA test

The immunologic hallmark of SLE is the presence of ANA. ANA is present in 95–99% of patients,^13^ so a negative test makes SLE unlikely. In children presenting with SLE symptoms but with negative ANA, lupus-like disorders such as monogenic interferonopathies and complement deficiencies should be considered.

Since ANA testing is highly sensitive, it comes at the cost of lower specificity as a trade-off. ANA is positive in approximately 5–10% of healthy adults,^14^ far exceeding the prevalence of SLE. ANA positivity can occur in a variety of other conditions, including autoimmune connective tissue diseases (CTD) (eg Sjogren’s disease, inflammatory myopathies and systemic sclerosis), viral infections and malignancy.

The gold standard for ANA test is indirect immunofluorescence assay (IFA), although multiplex immunoassays are increasingly used. Multiplex assays utilise beads coated with different antigens and have the advantages in detecting multiple specific autoantibodies in a single test, thus saving time and resources. These assays can yield false positive results up to 7%, particularly when low titres are detected,^15^ thus clinical correlation is important. In addition, low-level detection of lupus autoantigen on multiplex assay should be supplemented by ANA testing on IFA to improve sensitivity.

### Heterogeneity of SLE features

Early symptoms may be less specific, and several other medical conditions can mimic SLE. An example of an SLE mimicker is rosacea, which is often perceived as butterfly or malar rash. Malar rash is a photosensitive reddish or purplish rash that covers the cheeks and the bridge of the nose, typically sparing the nasolabial folds. While rosacea is a chronic inflammatory acneiform disease, often accompanied by episodes of flushing triggered by hot drinks, stress or alcohol. Thus, in the absence of systemic disease associated with facial rash, it may be appropriate to prioritise a dermatology referral.

Other less specific features and their mimickers are summarised in Table 2.

**Table 2:** Common less specific features of SLE and their mimickers.

Signs or symptoms	Mimickers
Butterfly or malar rash	Dermatology: rosacea, actinic keratosis, seborrhoeic dermatitis, contact dermatitis Infection: cellulitis, erysipelas, Lyme disease Autoimmune: dermatomyositis Metabolic: pellagra (niacin deficiency) Genetic: Bloom syndrome, homocystinuria
Mucosal ulceration	Physical: biting cheek, burns, poorly fitted dentures, smoking Inflammation/autoimmune: inflammatory bowel disease, coeliac, Behcet’s, lichen planus Metabolic: folate, B12 or iron deficiency Infection: HIV, herpes simplex virus, varicella zoster virus, hand, foot and mouth disease, syphilis, tuberculosis Psychological: stress, anxiety Iatrogenic: NSAIDs, beta-blocker, nicorandil Paraneoplastic: oral cancer
Hair loss	Genetic: male and female pattern baldness (androgenetic alopecia) Inflammation/autoimmune: alopecia areata Metabolic: iron, vitamin D, B vitamins, or zinc deficiency Hormone: thyroid disease, pregnancy, childbirth, menopause Infection: fungal infection Iatrogenic: chemotherapy, radiotherapy, methotrexate, anti-hypertensives (ACE inhibitor, beta-blocker), statins, anti-depressants, anti-convulsant, Parkinson’s disease drugs, acne drugs, warfarin Psychological: stress (telogen effluvium)
Raynaud’s phenomenon	Idiopathic: primary Raynaud’s Inflammatory/autoimmune: scleroderma, mixed connective tissue disease, Sjogren’s disease, inflammatory myopathies, rheumatoid arthritis, cryoglobulinaemia Physical: exposure to vibrating tools, smoking Iatrogenic: beta-blockers, anti-migraine drugs, decongestants Vascular/neurovascular: atherosclerosis, Buerger’s disease, primary pulmonary hypertension, thoracic outlet syndrome, carpal tunnel syndrome
Fatigue	Physical/lifestyle: poor sleep habits, lack of exercise, alcohol or substance misuse, stress and anxiety Hormone: thyroid disease, diabetes, adrenal insufficiency, menopause, vitamin D deficiency Chronic medical: anaemia of chronic disease, liver disease, heart failure, obstructive sleep apnoea, multiple sclerosis, Parkinson’s, myasthenia gravis Other chronic condition: chronic pain syndrome, fibromyalgia Inflammatory/autoimmune: autoimmune rheumatic diseases or connective tissue diseases, inflammatory bowel disease Infection: EBV or parovirus, COVID-19, post-viral infection, chronic hepatitis, HIV Iatrogenic: beta-blockers, benzodiazepines, tricyclic antidepressants, statins, opioid drugs, statins, chemotherapy
Myalgia (muscle pain)	Physical: repetitive strain injuries, traumatic injuries, sports injuries Inflammatory/autoimmune: inflammatory myopathies, Sjogren’s disease, scleroderma Neurology: neuromuscular diseases, muscular dystrophy Other chronic condition: chronic pain syndrome, fibromyalgia Iatrogenic: statins, vaccines, cocaine, occasionally ACE inhibitors Infection: Viral (influenza, COVID-19, HIV, EBV, enterovirus), bacteria (Lyme, pneumonia), dengue fever, malaria Metabolic/hormone: vitamin D deficiency, thyroid disease, electrolyte imbalance Paraneoplastic: sarcoma
Lymphadenopathy	Infections: viral (influenza, infective mononucleosis, HIV, measles), bacteria (*Streptococcus*, Lyme, typhoid), toxoplasmosis, syphilis, tuberculosis Inflammatory/autoimmune: sarcoidosis, Kikuchi disease, Rosai-Dorfman disease, Kimura disease Paraneoplastic: lymphoma, leukaemia, metastatic cancer

### Challenges in primary care

Due to early symptoms and signs being non-specific, ANA testing may be widely performed by GPs in situations with a low likelihood for SLE. Most positive results are in non-SLE pathology. Nevertheless, these positive tests usually lead to rheumatology referral. The clinical value of an ANA test is enhanced when there is a reasonable pre-test probability (ie clinical suspicion) of SLE or related CTD. However, non-specialists often have limited experience in identifying the most relevant clinical features, with only a handful of SLE patients in GP practice. Moreover, there are no evidence-based guidelines for GPs on when to request an ANA test and to refer to rheumatology.

### Challenges in secondary care

ANA-positive referrals with a low risk of SLE are a significant burden on secondary care and constitute up to 7% of rheumatologists’ new referral workloads.^16^ Once individuals with suspected SLE are referred to rheumatology, diagnosis remains challenging, since the cardinal symptoms or signs are usually not present at first referral.

It may be difficult for rheumatologists to confirm that potential features (eg rash) are autoimmune related without other investigations/opinions. For example, they may request a dermatology opinion and skin biopsy. Due to the complex multisystem nature of the disease, individuals with suspected SLE may require follow-up appointments and referral to different specialties for further tests and investigations. These result in an extended and expensive diagnostic process that only leads to SLE diagnosis or other CTD in 17% of new ANA-positive referrals from primary care, as reported in our ANA-positive clinic.^17^

### Social determinants of health

Emerging data show the impact of social determinants of health (SDH) on disparities in clinical presentation and outcomes in SLE. SDH is defined as ‘the condition in which people are born, grow, work, live, and age’. Health inequalities affecting the ethnic minorities, lower income level, lower educational attainment, neighbourhood crime, rural residence, and lower social support have been associated with severe SLE clinical presentation, disease activity and worse long-term outcomes.^18^

## Opportunities and future approach to improve SLE diagnosis

There are ample opportunities to intervene in the community, primary care and secondary/tertiary care environments to improve SLE diagnosis. By instilling awareness in the public, including people from the underserved groups, about SLE symptoms and encouraging open communication with healthcare professionals, work in communities can empower individuals to seek timely diagnosis and treatment.

In primary care, the rates of GP consultations and healthcare resources utilised by people with ultimate diagnosis of SLE increased exponentially in the years leading to diagnosis.^19^^,^^20^ Since ANA has very low specificity, a primary care risk score to predict a positive ANA that leads to appropriate referral to secondary care is warranted.

Beyond ANA, several diagnostic and prognostic biomarkers including interferon score, novel autoantibodies and proteomics are under development and validation. Once validated, these, together with other variables including polygenic risk scores, demographics and clinical characteristics, can be used to develop and validate a secondary care risk prediction model of progression to SLE from initial ANA-positive detection to allow rheumatologists to diagnose or exclude SLE earlier.

## Conclusion

Diagnosis of SLE remains challenging. A delay in diagnosis is associated with accrual of organ damage and worse disease outcomes, and negatively impacts quality of life. Classification criteria can be used as a guide, but they are not diagnostic. Increased awareness of this condition and better diagnostic tests in the future will facilitate rapid diagnosis, early intervention and trajectory of patients with SLE.

## CRediT authorship contribution statement

**Jean Scopes:** Writing – review & editing, Writing – original draft, Formal analysis, Methodology, Conceptualization, Visualization. **Md Yuzaiful Md Yusof:** Writing – review & editing, Writing – original draft, Formal analysis, Methodology, Conceptualization, Visualization, Supervision.

## Declaration of competing interest

The authors declare the following financial interests/personal relationships which may be considered as potential competing interests: Md Yuzaiful Md Yusof reports a relationship with Aurinia Pharmaceuticals Inc that includes: consulting or advisory. Md Yuzaiful Md Yusof reports a relationship with UCB that includes: consulting or advisory. Md Yuzaiful Md Yusof reports a relationship with Novartis that includes: board membership and speaking and lecture fees. Md Yuzaiful Md Yusof reports a relationship with GSK that includes: board membership. Md Yuzaiful Md Yusof reports a relationship with Alumis Inc. that includes: speaking and lecture fees. Md Yuzaiful Md Yusof reports a relationship with Roche that includes: speaking and lecture fees. Md Yuzaiful Md Yusof reports a relationship with Vifor Pharma Inc that includes: speaking and lecture fees. Jean Scopes reports a relationship with CESAS Medical Ltd that includes: speaking and lecture fees. If there are other authors, they declare that they have no known competing financial interests or personal relationships that could have appeared to influence the work reported in this paper.
